# Exploring transcriptomic signatures in sudden unexplained death (SUD) cases

**DOI:** 10.1007/s00414-025-03414-4

**Published:** 2025-02-21

**Authors:** Jacqueline Neubauer, Guro Dørum, Cordula Haas

**Affiliations:** 1 Zurich Institute of Forensic Medicine, University of Zurich, Zurich, Switzerland; 2 Nofima - Norwegian Institute of Food, Fisheries and Aquaculture Research, Ås, Norway

**Keywords:** Sudden unexplained death, Molecular autopsy, Transcriptome, Cardiac diseases

## Abstract

**Background:**

Molecular autopsy in sudden unexplained death (SUD) has successfully identified pathogenic variants in cardiovascular genes in a substantial proportion of cases, contributing to prevention strategies in family members. However, many SUD cases remain genetically unresolved, prompting investigations into other omics technologies to better understand the pathogenic mechanisms leading to a sudden death event. In this study, whole transcriptome sequencing was performed on heart samples from 43 SUD cases and 17 heart-healthy controls, with the aim to identify disease-specific transcriptome signatures in sudden unexplained death.

**Results:**

PCA based on the top 500 genes with the highest variance among the samples showed no clear separation between SUD and controls or among the three SUD subgroups. DESeq2 identified 1,676 differentially expressed genes between SUD and controls with significantly upregulated genes involved in biological processes such as angiogenesis, blood vessel development, vasculogenesis and cell adhesion. Pathway analysis of the differentially expressed genes showed that most were downregulated and involved in amide/peptide biosynthesis and fatty acid metabolism. Additional analysis of SUD subgroups revealed unique gene expression patterns and highlighted differentially expressed genes within each subgroup.

**Conclusion:**

Gene expression analysis of SUD heart tissue is a promising approach to identify cardiac disease-related pathways to further understand the pathological mechanisms leading to a sudden death event. However, due to the heterogeneity of the SUD cases and the unclear phenotype, further studies in larger cohorts are needed.

**Supplementary Information:**

The online version contains supplementary material available at 10.1007/s00414-025-03414-4.

## Introduction

Sudden cardiac death (SCD) is one of the leading causes of death worldwide [1]. While coronary artery disease (CAD) primarily accounts for SCD in the elderly, approximately 30% of sudden deaths under the age of 40 remain unexplained after thorough forensic autopsy procedures, with no clear cardiac etiology identified [2]. These cases are commonly referred to as sudden unexplained deaths (SUD) [3].

In recent years, post-mortem molecular autopsy in SUD has successfully identified pathogenic variants in genes associated with cardiovascular diseases in up to 30% of cases [4–9]. This approach has important implications for family members of the deceased, who may be at risk of similar cardiac conditions and for whom clinical and genetic evaluation could prevent another tragic sudden death [10, 11]. However, genetic testing alone is not always sufficient, as many SUD cases remain genetically unexplained. Therefore, continued exploration of other omics technologies, such as transcriptomics or proteomics, could improve our understanding of the pathogenesis of channelopathies and cardiomyopathies and might improve the accuracy of a diagnosis [12].

Myocardial transcriptome analysis in human cardiovascular diseases has shown that the expression pattern in cardiac tissue can be clearly distinguished between patients with various cardiac diseases such as arrhythmogenic right ventricular cardiomyopathy (ARVC) or dilated cardiomyopathy (DCM) compared to healthy individuals [13, 14]. Akdis et al. showed that myocardial mRNA expression profiles in the pathways involved in sarcolemma calcium regulation, apoptosis and adipogenesis are differentially expressed in patients with ARVC compared to DCM patients and controls, suggesting that these molecular pathways may play a critical role in the pathogenesis of ARVC [15]. In addition, transcriptome profiling of ACM (arrhythmogenic cardiomyopathy) patients with variants in the *FLNC* gene revealed dysregulated integrin-linked kinase and focal adhesion pathways, which may affect the actin cytoskeleton organization leading to impaired function and dysregulated signaling pathways [16]. In patients with hypertrophic cardiomyopathy (HCM), multi-omics analysis of hypertrophic pathways identified 8% differentially expressed genes (DEGs) between HCM and controls and a downregulation of canonical pro-hypertrophic signaling pathways, suggesting a late-state, cardioprotective attempt to circumvent hypertrophy [17]. In addition, clustering analysis of cardiac transcriptome profiles can help to identify specific cardiac diseases and subgroups, potentially contributing to personalized treatment strategies and improved prognostic accuracy for patients [18, 19].

To date, only one study has analyzed the heart transcriptome in SUD cases. Andersen et al. performed whole genome sequencing (WGS) and whole transcriptome sequencing (WTS) in sudden arrhythmic death syndrome (SADS) victims in order to correlate gene expression levels with DNA variants in regulatory non-coding regions of the genome [20]. In a cohort of nine SADS and four SUDI (sudden unexplained death in infancy) cases, they identified a rare variant in the promoter region of *NEXN* (c.-194 A > G), that was statistically significantly associated with reduced *NEXN* expression and cardiac hypertrophy.

In this study, we performed a systematic whole transcriptome analysis in 43 SUD cases and 17 controls to identify potential disease-specific transcriptome signatures. The samples were analyzed with DESeq2 to identify differentially expressed (DE) genes between SUD cases and controls, and the DE genes were further subjected to KEGG pathway and GO analysis to better understand the functions of the genes involved. To account for the heterogeneity of different diseases within our SUD cohort, cases were divided into three subgroups based on detailed post-mortem examination of the heart at both microscopic and macroscopic levels. We repeated the DESeq2, KEGG pathway and GO analysis for each of the three subgroups to investigate how the transcriptomic signatures varied between them.

## Materials and methods

### SUD cohort

Between 2012 and 2020, 55 unrelated cases of sudden unexplained death were autopsied at the Zurich Institute of Forensic Medicine (ZIFM) in Switzerland [8, 21]. All SUD victims were examined according to European and Swiss guidelines for the management of young SUD cases [22–24], including a death scene investigation, a complete autopsy examination with pharmacological-toxicological and histopathological screening, and a review of the clinical history. The standard heart examination included a macroscopic inspection of the external structures of the heart and assessment of the anatomy of the great arteries, followed by the opening of the heart. The valves as well as the ventricular thickness were measured and the musculature was examined in a cross-section through the anterior and posterior walls, including the septum of the left ventricle. In addition, the total weight of the heart was assessed according to Zeek [25]. For all cases, a second expert opinion was obtained from cardio-pathologists and cardiologists at the University Hospital Zurich. By definition, the general cut-off age for SUD is 40 years of age, but older autopsy-negative sudden death cases were included in the study cohort if a cardiac disease was suspected in the absence of any evidence of myocardial infarction [26]. Shock-frozen heart tissue was available for 43 (mean ± SD, 32.3 ± 12.2 years of age; range, 6–55 years of age) of the 55 SUD cases. The SUD cohort of 43 individuals consisted of 30 males (mean ± SD, 33.2 ± 10.8 years of age; range, 6–55 years of age) and 13 females (mean ± SD, 29.5 ± 14.5 years of age; range, 6–51 years of age). Five SUD victims were younger than 18 years of age, including three females (aged 6, 8, and 11 years) and two males (aged 6 and 17 years). Most SUD victims were of European origin (88.3%). A further five cases (11.7%) were of African origin. Postmortem exome sequencing (WES) with a focus on cardiac- and epilepsy-associated genes has been performed in this cohort in previous studies [8, 21, 27]. A detailed summary of all cases is provided in Supplementary Table S1.

### Controls

The control group consisted of 17 heart-healthy individuals who had died suddenly (mean ± SD, 28.6 ± 16.7 years of age; range, 4–66 years of age), including 13 males (mean ± SD, 30.6 ± 18.3 years of age; range, 4–66 years of age) and four females (mean ± SD, 22.3 ± 6.3 years of age; range, 17–33 years of age) (Supplementary Table S2). The majority of controls were of European origin (88%), with one case of African origin and one of Indian origin. Causes of death included accidents, suicide, intoxication and non-cardiac conditions such as stomach ulcer or meningitis.

### Tissue collection and RNA extraction

During autopsy, small pieces of the left ventricular free wall were collected, immediately shock-frozen in liquid nitrogen and stored at -80° C. Small pieces of heart tissue (approximately 100 mg) were cut and homogenized in 1-Thioglycerol-Homogenization solution. Tissue homogenization was performed using the Bead Ruptor Elite (Omni International, Kennesaw, Georgia, USA) or the Precellys Evolution (Bertin Instrument, Montigny-le-Bretonneux, France). Tissue was homogenized with CK14 soft tissue beads (Fisher Scientific) for 20s at 5500 rpm for two cycles with a 30s pause between the cycles or until no visible tissue pieces remained. RNA extraction of homogenates was performed using the Maxwell^®^RSC simplyRNA cells and simplyRNA tissue Kit (Promega, Madison, Wisconsin, USA) according to the manufacturer’s protocol. RNA was quantified with a Quantus™ fluorometer (Promega) and RNA integrity (RIN) was measured using an Agilent 2200 TapeStation (Agilent Technologies Inc., Santa Clara, California, USA).

### RNA sequencing and bioinformatic analysis

RNA library preparation was performed using the Trio RNA-Seq Kit (Tecan, California, USA), including rRNA depletion according to the manufacturer’s protocol. For the library preparation, 50 ng of total RNA or a maximum of 10 µl if less RNA was available, was used as input. Library amplification was done using six amplification cycles. The library quality was checked with the Agilent 2200 TapeStation. A pre-sequencing quality control run was performed on an Illumina MiniSeq (Illumina, San Diego, California, USA). The settings were 75 bp fragment length paired-end sequencing with 100,000 reads for each sample. Final RNA sequencing was done on an Illumina NovaSeq 6000 at the Functional Genomics Center Zurich, University of Zurich, Switzerland, performing single-end sequencing with a synthesis of 100 cycles using an SP flow cell (batch 1) and an S1 flow cell (batch 2).

After sequencing, reads were quality-checked using FastQC. Sequencing adapters were removed with Trimmomatic and reads were hard-trimmed at 5 bases at the 3’ end [28]. Subsequently, reads of at least 20 bases in length, and with an overall average Phred quality score greater than 30, were aligned to the Homo Sapiens reference transcriptome (FASTA and GTF files, respectively, downloaded from GRCh38) using STAR v.2.5.1 with default settings for single-end reads [29]. The distribution of the reads across genomic isoform expression was quantified using the R package *GenomicRanges* from Bioconductor version 3.0 [30]. In addition, the sequence classification tools SILVA [31] and Kraken [32] were used to align reads against multiple non-human transcriptomes (e.g. bacterial or viral species) to avoid incorrect inferences due to possible contamination or post-mortem effects [33, 34]. The CountQC application from the SUSHI framework was used to check the gene-level counts [35]. Each sample was required to have genomic features with reads above 30% to qualify for further analysis.

### Normalization

Data were pre-filtered by requiring genes to have a minimum of 10 counts for at least seven samples (equal to the number of samples in the smallest SUD subgroup) to be included for further analysis. Prior to any exploratory data analysis, read counts were normalized with a variance stabilizing transformation (VST) from DESeq2 [36], which produces transformed data on the log2 scale normalized with respect to library size.

### Differential expression analysis and gene set analysis of the transcriptome data

Differential expression (DE) analysis was performed on raw read counts using the R package DESeq2 version 1.40.2 [36]. Sex, age (categorized as below or above 18 years) and batch (1or 2) were included as known covariates in the analysis. Wald test p-values were computed for the comparison of SUD and controls, and false discovery rates (FDR) were calculated to adjust for multiple hypothesis testing. Genes with an FDR < 0.1 and log2 fold change > 0.5 were assigned as significantly differentially expressed.

DEGs were interpreted using the Kyoto Encyclopedia of Genes and Genomes (KEGG) (https://www.genome.jp/kegg/), a free database resource containing a variety of biochemical pathways [37]. In addition, Gene Ontology (GO) analysis was used to assess biological processes, molecular functions and cellular components represented among the DEGs. The KEGG and GO analyses were performed with the R package *gage* [38], with an FDR ≤ 0.1 set as the threshold for significant pathways/groups. Finally, we compared our list of DEGs to a list of 278 previously identified cardiac related genes (Supplementary Table S3) [8, 39].

## Results

### Study cohorts

Fresh-frozen heart tissue from the left ventricle was used for the transcriptome analysis of 43 SUD and 17 heart-healthy deceased controls. RNA library preparation and sequencing were successfully performed on all samples. On average, 35.1 ± 10.6 million reads were obtained per sample. Approximately 61% of the sequence reads were uniquely mapped to the human reference genome GRCh38. As a quality criterion, samples were required to have reads above 30% of genomic features, which was not met for eight SUD samples, leaving a total number of 35 SUD and 17 control samples for differential transcriptome analysis (Supplementary Tables S1 and S2).

By definition, SUD encompasses a range of diverse pathological mechanisms, resulting in a highly heterogeneous study cohort. Therefore, the SUD cases were further divided into three subgroups according to the heart weight [25], as well as microscopic and macroscopic findings of the heart. The “*primary normal”* subgroup included 16 cases (12 males / four females; mean ± SD, 28.9 ± 11.75 years of age) with a normal heart weight and no other abnormalities in the heart, most likely representing arrhythmias. A further 12 cases (five males / seven females; mean ± SD, 36.1 ± 11.9 years of age) with an enlarged heart and / or other mild cardiac abnormalities (hypertrophic cardiomyocytes, discrete fibrosis, mild fatty deposits) were labelled as “*primary cardiomyopathy*”. Cases with signs of a cardiac disease and secondary factors such as hypertension or fibrosis (but not sufficient to explain the occurrence of sudden death), were grouped into “*secondary condition*” (n = seven; five males / two females; mean ± SD, 36.5 ± 8.04 years of age). Sudden unexpected death in epilepsy (SUDEP) was considered the most likely cause of death in seven (four males / three females; mean ± SD, 26.1 ± 8.8) of the 35 SUD cases, according to the definition by Nashef et al. [40]. Several mechanisms, including dysregulation of the cerebral and autonomic nervous systems, respiratory dysfunction, and cardiac abnormalities during and between seizures, may contribute to the pathogenesis of SUDEP [41]. However, as only heart tissue and no other tissue samples (e.g. brain tissue) were available for RNA sequencing, SUDEP cases were not considered as a separate group, and were therefore classified into the above-mentioned subgroups according to the findings of the detailed examination of the heart.

### Normalization and exploratory data analysis

**Fig. 1:**
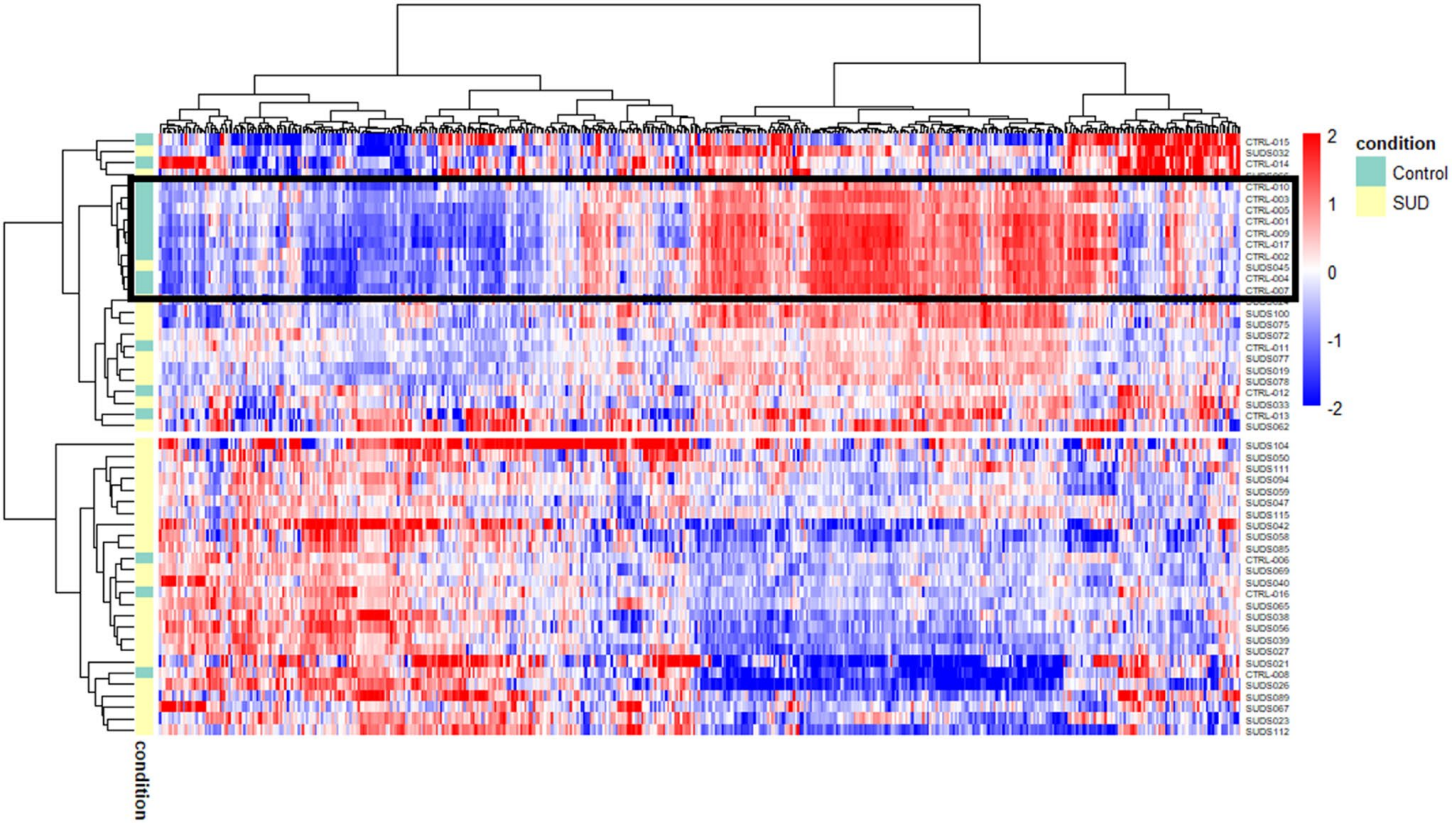
Heatmap of the top 500 genes with the highest variance. The black frame displays a cluster of samples with a distinct expression pattern, including nine controls that suffered a fast death and one SUD. Red-blue scale: red represents upregulation and blue downregulation

The raw read count files of the SUD and control cohorts contained counts for a total of 19,466 genes. After pre-filtering, 13,226 genes were retained for further downstream analysis. The heatmap in Fig. 1 shows the VST-transformed read counts for the 500 genes with the highest variance across all samples. The samples are divided into two main clusters: the upper main cluster (*n* = 26) contains a mix of SUD and control samples, while the bottom main cluster (*n* = 26) contains primarily SUD cases in addition to three control cases. The upper main cluster is further divided into three sub-clusters. The black frame highlights an interesting sub-cluster of nine controls and one SUD sample showing a distinct expression pattern. Closer examination of the expert reports revealed that these control samples were from individuals that suffered a fast death (e.g. accidents, suicide), whereas the remaining control samples were from people who died more slowly (such as anaphylactic shock, intoxication, etc.). Interestingly, one SUD case (SUDS045), classified as “*primary cardiomyopathy*”, also clustered closely with these nine control samples. SUDS045 was a 50-year-old woman whose death was not witnessed and whose microscopic and macroscopic examination of the heart showed extensive fatty infiltration of the right ventricular wall. The other two sub-clusters in the upper main cluster are composed of SUD and control cases with no obvious similarities (age, batch, sex) that could explain a similar expression pattern (Supplementary Figure S1). In the bottom main cluster, SUDS104, classified as “*primary normal*”, forms a separate sub-cluster compared to all other samples in this cluster. SUDS104 is a 17-year-old male with a diagnosed SQT syndrome and a known family history of SCD in several relatives. In our previously published study [21], postmortem genetic testing in SUDS104 identified a variant in the *SLC4A3* gene (c.3117C > A, p.(Ser1039Arg)), and cardio-genetic counselling of close relatives revealed additional phenotype positive family members carrying the same *SLC4A3* variant. For the remaining SUD and control samples in the bottom main cluster, there is no apparent separation according to age, batch, sex, postmortem interval (PMI) and ethnicity (Supplementary Figure S1).

**Fig. 2:**
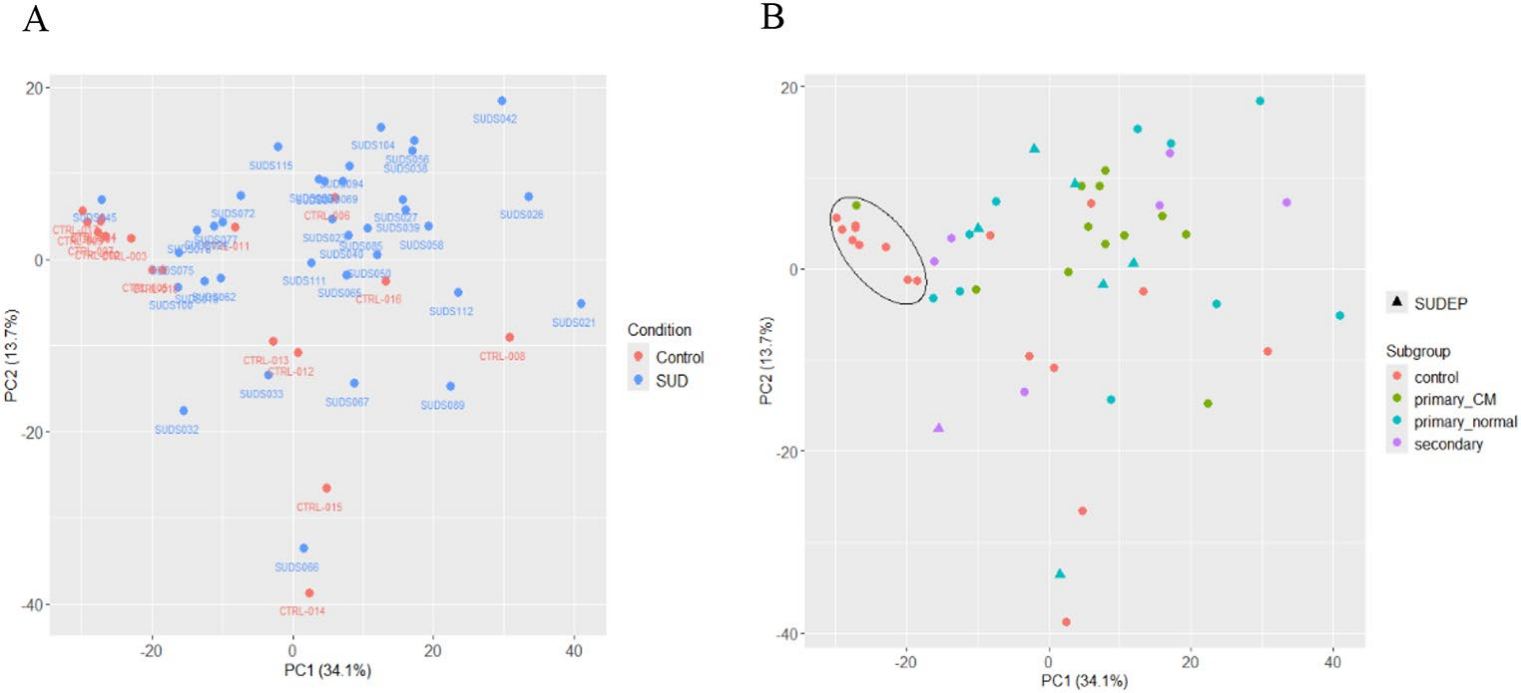
PCA plots of the top 500 genes with highest variance among the 35 SUD and 17 control cases, displaying the first two principal components. **A**) Samples are separated according to SUD and control, labelled with the sample name. **B**) SUD samples are divided into the subgroups “*primary normal*”, “*primary cardiomyopathy*” (primary_CM) and “*secondary condition*” (secondary). In addition, the seven SUDEP cases are labelled with a triangle symbol. The samples in the black ellipse are control samples that suffered a fast death and one SUD case (SUDS045)

In the PCA plot based on the top 500 genes with the highest variance among the samples, no separation was observed between the 35 SUD and 17 control cases along the first two components (Fig. 2A). No clear groupings can be seen for the three SUD subgroups (“*primary normal”*, “*primary cardiomyopathy”*, “*secondary condition”*) either (Fig. 2B). In addition, no clustering was observed for the seven cases either with a known medical history of epilepsy or where SUDEP was considered the most likely cause of death in the absence of any previously reported epileptic events. A close-knit cluster can be observed for the control individuals who suffered a fast death (Fig. 2B, marked by an ellipse). In addition, PMI, batch, sex, age, and ethnicity did not appear to affect the gene expression profiles of the top 500 genes (Supplementary Figure S2). In Supplementary Figure S2D, the samples are divided into three age categories: below 18, 18–40 and above 40 years of age. Although there is no apparent separation between the samples below and above 18 years, there could still be some age effect, therefore PCA was repeated using only individuals over 18 years to see how this would affect the analysis. The corresponding PCA plot is shown in Supplementary Figure S3. The first component explains 42% of the variation in the data, compared to 34% in the PCA including the young samples; but no new patterns can be detected.

Because of a visible difference in the control cases that suffered a fast death (Figs. 1 and 2), we decided to divide the control samples into two subgroups: “*fast death*” (*n* = nine; five males / four females; mean ± SD, 25.3 ± 16.24 years of age) and “*other*” (*n* = eight; eight males; mean ± SD, 32.3 ± 16.41 years of age). Since our goal is to compare the SUD cases to all control cases rather than just one subgroup, this division was made solely to account for unwanted variation in the DESeq2 analysis, providing more accurate results.

### Gene expression differences between SUD and control samples

For the differential expression analysis with DESeq2, we compared the three SUD subgroups (*“primary normal”*, “*primary cardiomyopathy” and* “*secondary condition”)* with the two control subgroups (“*fast death*” and “*other*“). To adjust for unequal sample sizes between subgroups, a contrast was applied that assigned weights to each subgroup based on its size.

**Fig. 3:**
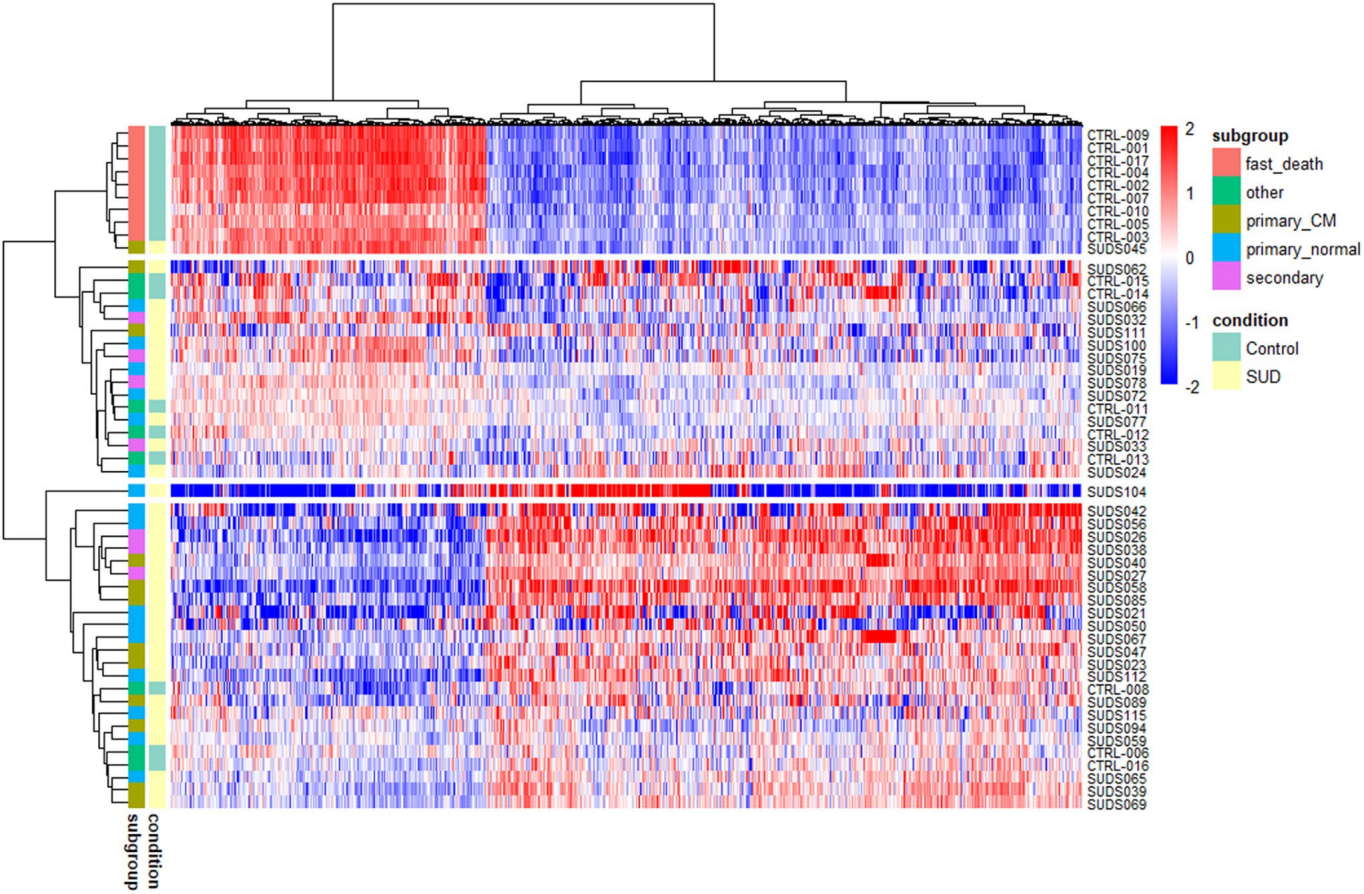
Heatmap of 1,676 significantly differentially expressed genes between SUD and controls. Samples were annotated to subgroups within SUD (“*primary normal*”, “*primary cardiomyopathy*” (primary_CM) and “*secondary condition*” (secondary)) and controls *(“fast death”* and *“other”*). Red-blue scale: red represents upregulation and blue downregulation

DESeq2 identified 1,676 genes (13%) significantly differentially expressed between SUD and control cases (Fig. 3), of which 1,093 are upregulated and 583 are downregulated in SUD cases. The cluster containing the “*fast death*” control samples and one SUD sample (SUDS045, “*primary cardiomyopathy*”) shows a very distinct expression pattern for the DE genes. For the rest of the SUD and control cases, no clustering between the different subgroups and control cases was observed.

**Fig. 4:**
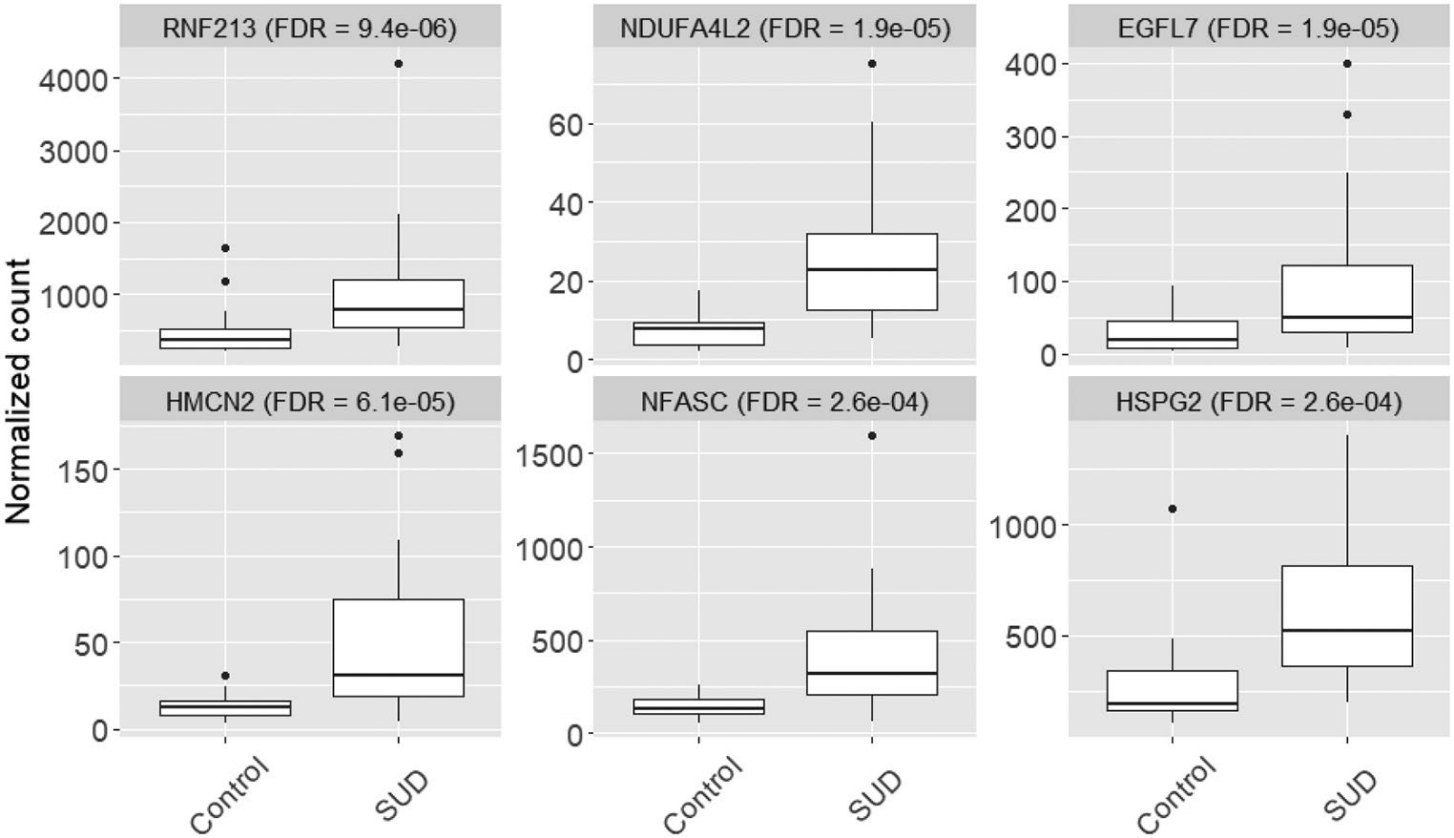
Boxplot of normalized read counts in SUD and control samples for the top six DE genes. All genes are upregulated in the SUD samples

Figure 4 shows the normalized read counts in the SUD and the control cases for the six most differentially expressed genes: *RNF213* (FDR = 9.4e-06), *NDUFA4LD* (FDR = 1.9e-05), *EGFL7* (FDR = 1.9e-05), *HMCN2* (FDR = 6.1e-05), *NFASC* (FDR = 2.6e-04) and *HSPG2* (FDR = 2.6e-04). All six genes are upregulated in the SUD cases and are involved in biological processes such as angiogenesis (*RNF213*,* EGFL7*,* HSPG2*), blood vessel development (*EGFL7*), vasculogenesis (*EGFL7*), cell adhesion (*EGFL7*,* HMCN2*,* NFASC*) and positive regulation of endothelial cell proliferation (*HSPG2*).

**Fig. 5:**
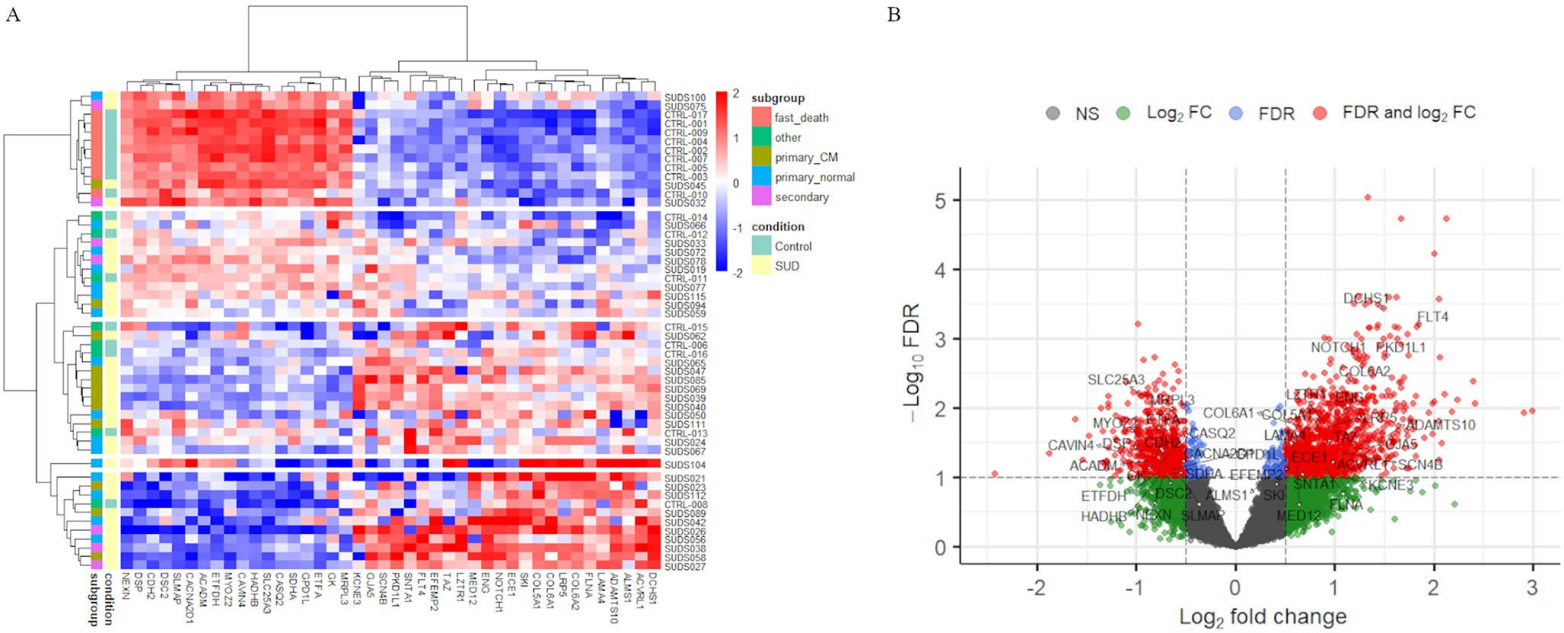
Gene expression differences between SUD and control samples with a focus on cardiac related genes. **A**) Heatmap of the 42 cardiac genes significantly differentially expressed between SUD and control samples. Red-blue scale: red represents upregulation and blue downregulation. **B**) Volcano plot showing the log2 fold change plotted against the FDR for each gene tested in DESeq2. Differentially expressed genes that have both FDR ≤ 0.1 and a log fold change ≥ 0.5 are marked in red. The 42 significant genes linked to cardiac diseases are labelled. The most upregulated genes are towards the right, the most downregulated genes are towards the left, and the most statistically significant genes are towards the top.

 Among the 1,676 genes identified as differentially expressed between SUD and control samples, 42 are present in our list of candidate genes associated with cardiovascular diseases (labelled in the volcano plot in Fig. 5B, and listed in Supplementary Table S4). Of these, 26 are upregulated and 16 are downregulated. In a heatmap of these 42 cardiac related DE genes (Fig. 5A), the cluster containing the “*fast death*” control samples shows a very distinct expression pattern, together with four SUD cases (SUDS032, SUDS045, SUDS075 and SUDS100) from different subgroups. The rest of the SUD and control cases cluster within four sub-clusters without any obvious association based on the three SUD subgroups. Again, SUDS104 forms a separate sub-cluster.

**Fig. 6:**
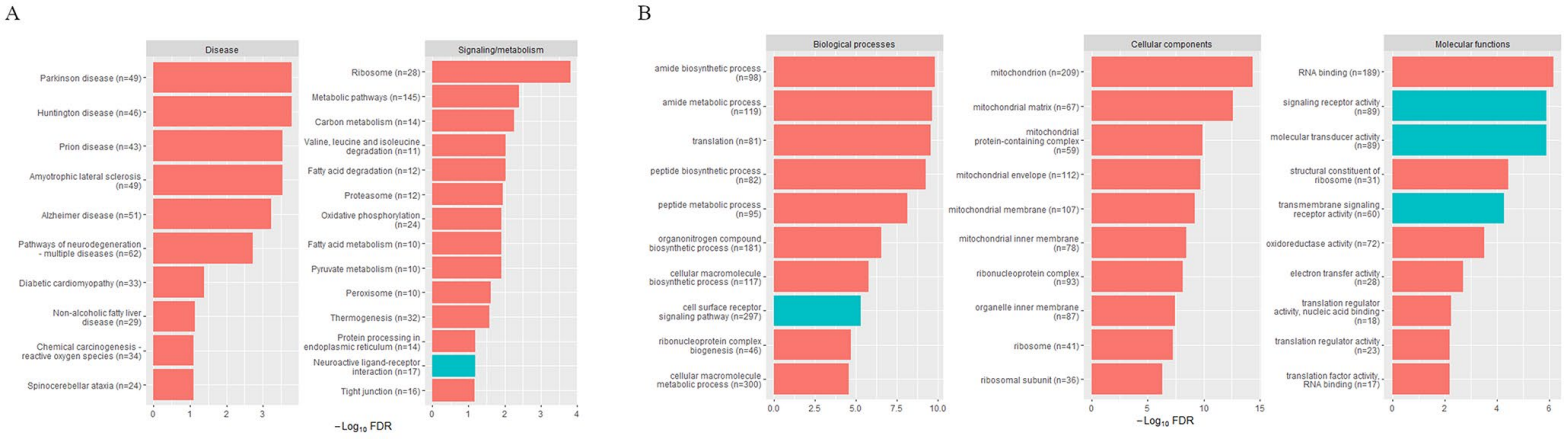
**A**) Significant KEGG pathways among genes differentially expressed in SUD cases compared to controls (FDR ≤ 0.1), divided into “Disease” and “Signaling/metabolism pathways” and **B**) the top 10 differentially expressed GO terms (FDR ≤ 0.1) in each of the three categories biological processes, cellular components and molecular function. Red bars represent downregulated terms and green represent upregulated terms. The effective gene set size used in the analysis is indicated by n

KEGG pathway analysis of the 1,676 differentially expressed genes identified one upregulated and 23 downregulated pathways in SUD cases compared to controls (Fig. 6A). The GO enrichment analysis revealed 216 upregulated and 103 downregulated biological processes, 8 upregulated and 20 downregulated molecular functions, and 7 upregulated and 54 downregulated cellular components in SUD cases compared to controls (Fig. 6B).

### Investigation of SUD subgroups

To further investigate where the differences between SUD and control cases might be found, we examined the individual SUD subgroups. Although no clear division into subgroups could be observed in the PCA plot in Fig. 2, the differential expression analysis with DESeq2 was repeated, this time comparing the individual SUD subgroups with the control cases, presented in the following sections.

#### “Primary normal” subgroup

**Fig. 7:**
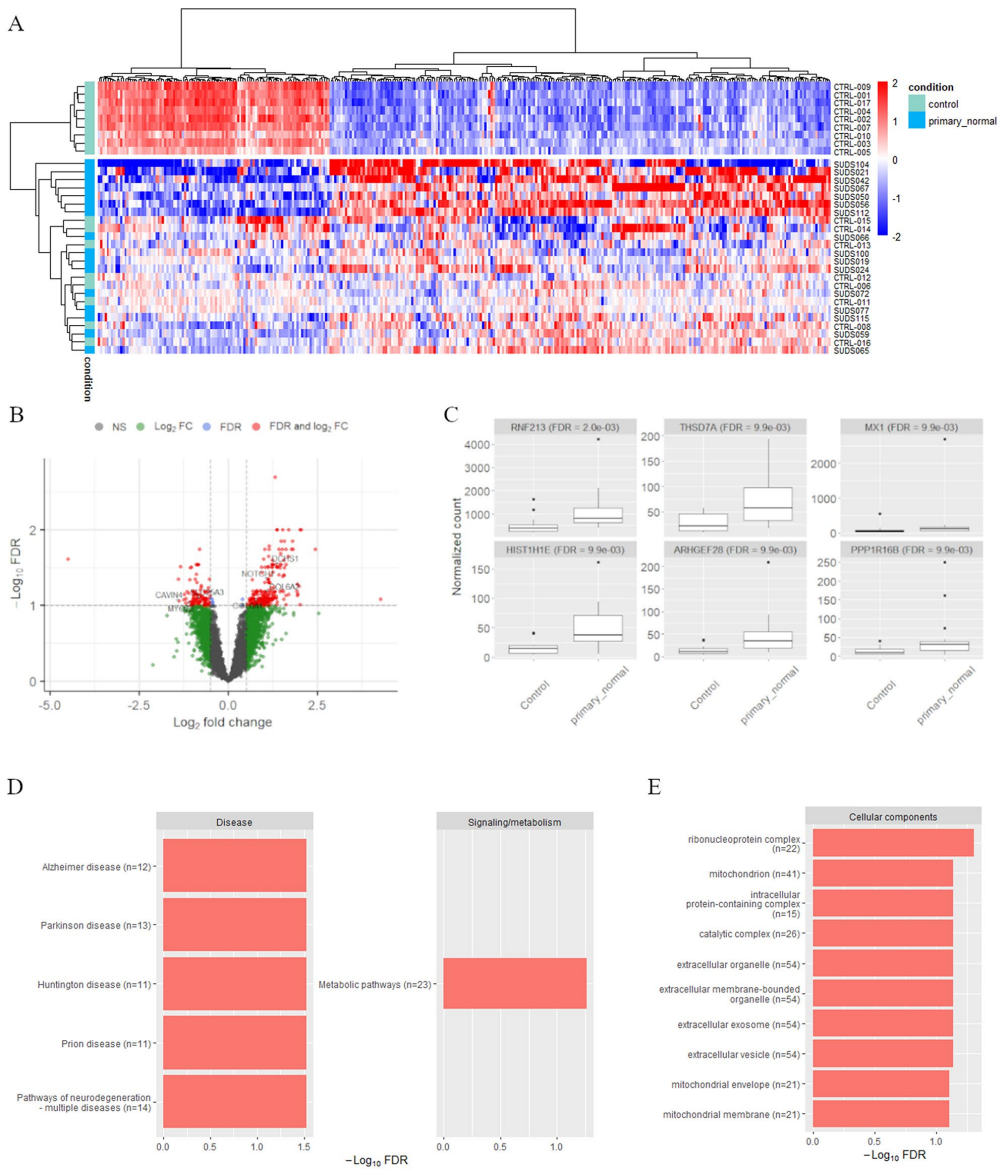
Gene expression differences between “*primary normal*” and control samples. **A)** Heatmap of the 332 significantly differentially expressed genes. Red-blue scale: red represents upregulation and blue downregulation. **B)** Volcano plot with DE genes that have both FDR ≤ 0.1 and a log fold change ≥ 0.5 marked in red. The seven significant cardiac genes are labelled. The most upregulated genes are towards the right, the most downregulated genes are towards the left, and the most statistically significant genes are towards the top.** C)** Boxplot of normalized read counts for the top six most differentially expressed genes. Only one gene (*RNF213*) overlaps with the genes in Fig. 4 comparing all SUD and control samples. **D)** Significant KEGG pathways (FDR ≤ 0.1) among the DE genes in the categories “Disease” and “Signaling/metabolism” and **E)** significant GO terms (FDR ≤ 0.1). Significant GO terms were only found in the category cellular components. Red bars represent downregulated terms. The effective gene set size n is indicated

The comparison of the 16 “*primary normal*” cases against the control cases yielded 332 significant genes, with 224 upregulated and 108 downregulated genes (Fig. 7A). The cluster containing the “*fast death*” control samples shows a very distinct expression pattern, whereas the rest of the controls and all 16 “*primary normal*” cases cluster together. Of the 332 significant DE genes, seven also appear in our list of cardiac related genes (Fig. 7B and Supplementary Table S5). Figure 7C shows the read counts for the six most differentially expressed genes: *RNF213* (FDR = 2.0e-03), *THSD7A* (FDR = 9.9e-03), *MX1* (FDR = 9.9e-03), *HIST1H1E* (FDR = 9.9e-03), *ARHGEF28* (FDR = 9.9e-03) and *PPP1R16B* (FDR = 9.9e-03). All six genes are upregulated in the “*primary normal*” group and are involved in biological processes such as angiogenesis (*RNF213*,* THSD7A)* or apoptotic processes (*MX1)*. Only one of the genes, *RNF213*, is among the top six genes that also showed differential expression between all SUD and controls (Fig. 4).

KEGG pathway analysis of the 332 DE genes yielded five downregulated disease pathways and one downregulated signaling and metabolism pathway, while GO analysis revealed ten significant downregulated cellular components and no significant biological processes or molecular functions (Fig. 7D and 7E).

#### “Primary cardiomyopathy” subgroup

**Fig. 8:**
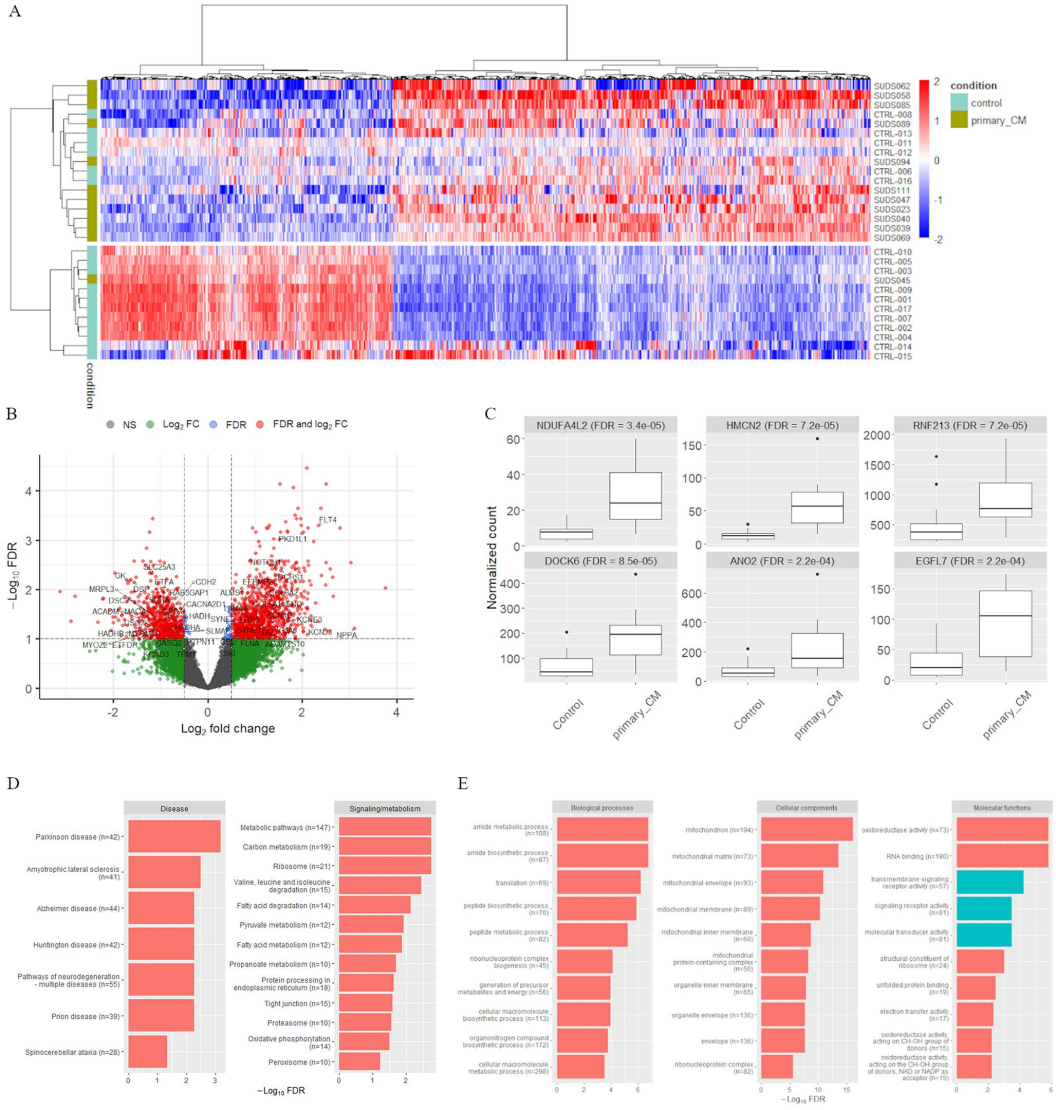
Gene expression differences between *“primary cardiomyopathy”* and control samples. **A**) Heatmap of the 1,652 significantly differentially expressed genes. Red-blue scale: red represents upregulation and blue downregulation. **B**) Volcano plot with DE genes that have both FDR ≤ 0.1 and a log fold change ≥ 0.5 marked in red. The 48 significant cardiac genes are labelled. The most upregulated genes are towards the right, the most downregulated genes are towards the left, and the most statistically significant genes are towards the top. **C**) Boxplot of normalized read counts for the top six DE genes. Four of the six genes overlap with the genes in the boxplot in Fig. 4 comparing all SUD and control samples. **D**) Significant KEGG pathways (FDR ≤ 0.1) in the categories “Disease” and “Signaling/metabolism” and **E**) the top 10 significant GO terms (FDR ≤ 0.1) in the categories biological processes, cellular components and molecular functions. Red bars indicate terms that are downregulated in “*primary cardiomyopathy*”, while green bars indicate upregulated terms, and n is the gene set size

For the comparison of the 12 “*primary cardiomyopathy*” cases against the controls, 1,652 genes were found to be significantly differentially expressed, of which 1,025 were upregulated and 627 were downregulated (Fig. 8A). The “*fast death*” control samples and one SUD case (SUD045) show a very distinct expression pattern, whereas the rest of the controls and all 12 “*primary cardiomyopathy*” cases cluster together. Among the 1652 DE genes, 48 were also found in our list of cardiac related genes (Fig. 8B and Supplementary Table S6). The top six DE genes are shown in Fig 8C: *NDUFA4L2* (FDR = 3.4e-05), *HMCN2* (FDR = 7.2e-05), *RNF213* (FDR = 7.2e-05), *DOCK6* (FDR = 8.5e-05), *ANO2* (FDR = 2.2e-04) and *EGFL7* (FDR = 2.2e-04). All six genes are upregulated in the SUD cases. *RNF213* was one of the top six genes exhibiting differential expression both between all SUD and controls and between “*primary normal*” and controls (Figs. 4 and 7C).

The 1,652 DE genes were further subjected to KEGG and GO analysis. The KEGG analysis revealed 13 significantly downregulated signaling and metabolism pathways and seven downregulated disease pathways (Fig. 8D). The GO analysis showed one upregulated and 94 downregulated biological processes, eight upregulated and 22 downregulated molecular functions and two upregulated and 52 downregulated cellular components (Fig. 8E).

#### “Secondary condition” subgroup

**Fig. 9:**
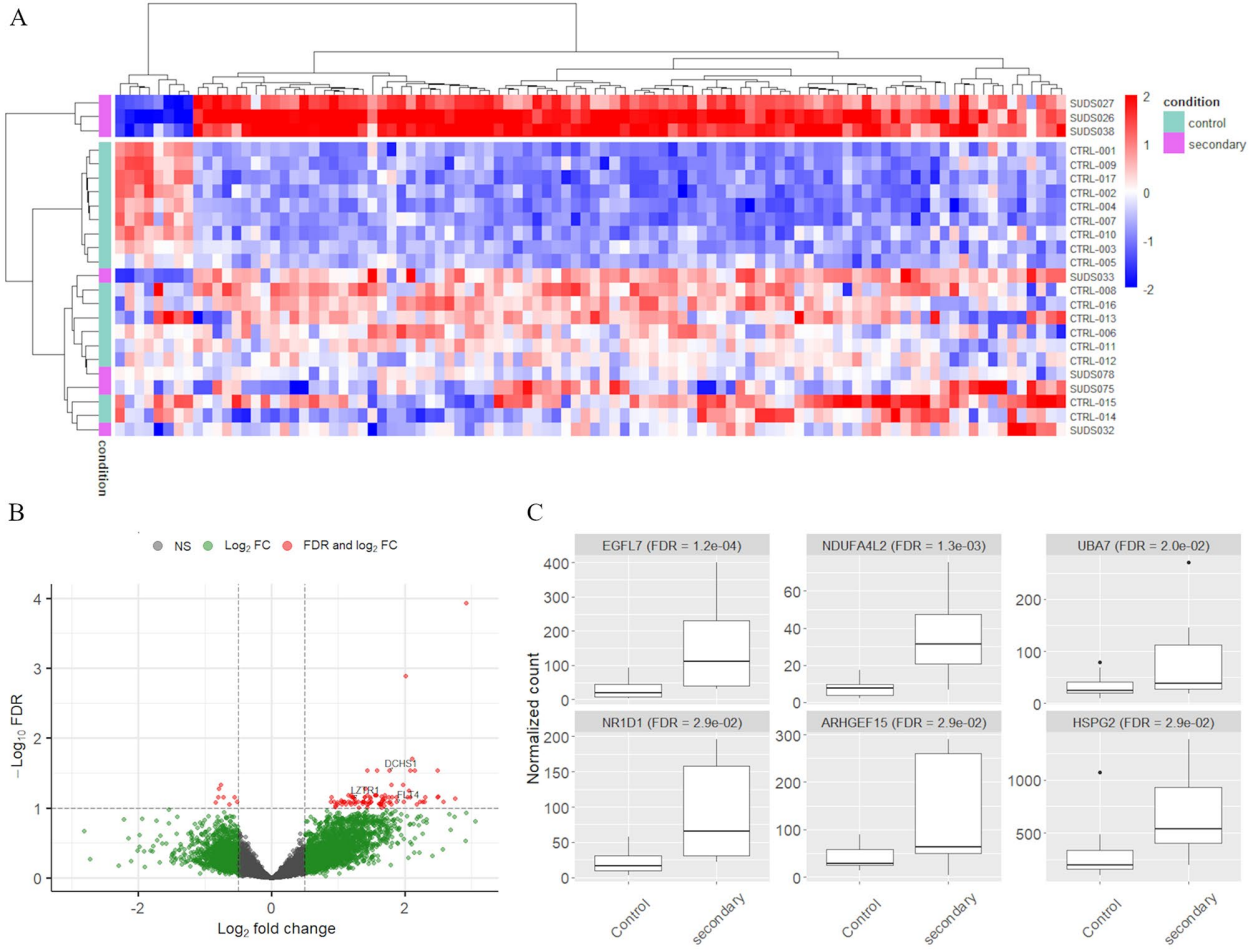
Gene expression differences between *“secondary condition”* and control samples. **A**) Heatmap of the 98 significantly differentially expressed genes. Red-blue scale: red represents upregulation and blue downregulation. **B**) Volcano plot with DE genes that have both FDR ≤ 0.1 and a log fold change ≥ 0.5 marked in red. The three significant cardiac genes are labelled. The most upregulated genes are towards the right, the most downregulated genes are towards the left, and the most statistically significant genes are towards the top. **C**) Boxplot of normalized read counts for the top six DE genes. Three of the six genes overlap with genes in the boxplot in Fig. 4 comparing all SUD and control samples

Finally, the comparison of the “*secondary condition*” cases (*n* = 7) and the control cases yielded 98 significantly differentially expressed genes, with 90 upregulated and eight downregulated genes (Fig. 9A). Interestingly, three SUD cases (SUDS026, SUDS027 and SUDS038) form a very distinct cluster. Closer examination of the expert reports of these cases revealed no known common characteristics that could explain their separation from the rest of the SUD cases. In the second cluster, the “*fast death*” control samples form a separate sub-cluster from the remaining four SUD and control cases. Of the 98 DE genes, three are also included in our list of cardiac related genes (Fig. 9B and Supplementary Table S7). The read counts for the top six most differentially expressed genes are shown in Fig 9C: *EGFL7* (FDR = 1.2e-04), *NDUFA4L2* (FDR = 1.3e-03), *UBA7* (FDR = 2.0e-02), *NR1D1* (FDR = 2.9e-02), *ARHGEF15* (FDR = 2.9e-02) and *HSPG2* (FDR = 2.9e-02). Although there are few DE genes, the boxplot shows that the most significant genes show a clear distinction between *“secondary condition”* and control samples. All six genes are upregulated in the SUD cases and three of them (*EGLF7*,* NDUFA4L2* and *HSPG2)* are also among the top six genes differentially expressed between all SUD and controls (Fig. 4). In addition, two genes (*EGLF7* and *NDUFA4L2)* are identified among the top six in the “*primary cardiomyopathy*” subgroup (Fig. 8C).

The KEGG and GO analysis of the 98 DE genes resulted in no significant pathways and GO terms.

#### Comparison of subgroup results

The Venn diagrams in Supplementary Figures S4 and S5 show the overlapping DE and cardiac genes among the different subgroups, as well as between individual subgroups and all SUD cases. All the subgroups have some unique DE genes. The primary cardiomyopathy samples show the highest number of unique genes, but is also the subgroup with the highest number of DE genes (Supplementary Figure S4). All three subgroups have at least one differentially expressed cardiac gene that is unique for the group (Supplementary Figure S5A).

#### Expression patterns of cardiac disease-associated genes with pathogenic or likely pathogenic variants within the SUD cohort

Previously performed WES analysis of the SUD cases had identified likely pathogenic or pathogenic variants in 13 genes associated with cardiac diseases (Supplementary Table S1) [8, 21]. The boxplots in Supplementary Figure S6 display normalized read counts for these 13 genes in SUD and control samples, with each gene having at least one sample that exhibits a pathogenic or likely pathogenic variant. These samples with a variant do not appear to have extreme read counts overall.

## Discussion

Sudden unexplained death in young people is a devastating event, and although postmortem molecular autopsy can help to identify underlying genetic diseases in some of the cases, extending research to other omics technologies such as transcriptomics can provide a deeper understanding of the pathophysiological mechanisms leading to sudden death.

In this study, we conducted a systematic whole transcriptome analysis of 43 SUD cases and 17 controls. By comparing the RNA expression patterns between these two cohorts, we aimed to uncover distinct molecular patterns that might provide insights into the underlying mechanisms leading to a sudden death event. Heart tissue was used for RNA sequencing, as it is the most promising tissue to investigate impaired pathways, given that SUD is often associated with specific cardiac diseases [42]. Overall, post-mortem tissue samples provide a valuable source for studying different disease mechanisms in specific organs, as sampling from living individuals would be impossible. However, it is important to note that RNA expression levels in postmortem tissue can be affected by temperature at the time of death, storage conditions, tissue type, biological responses to death and/or by the PMI, which is defined as the interval between death and sample collection [43]. RNAs from highly expressed genes may persist for a longer time, whereas RNAs from lowly expressed genes may degrade more rapidly [44]. Consequently, the expression levels of RNAs may be skewed, particularly for low abundance genes. The PMI in our SUD and control cases showed a rather large variability of up to 78 h (mean ± SD: 29.7 ± 15.2 h; min = 9 h), but no clustering was observed when separating the samples into short (< 10 h), medium (11–24 h) and long (> 24 h) PMI. An important measure of the quality of RNA samples is the RNA integrity number (RIN) [45]. According to this quality assessment, all of our analyzed RNA samples were highly degraded (RIN-values between 1 and 6), which could lead to a loss of intact full-length transcripts and consequently to skewed gene expression profiles or an underrepresentation or absence of certain genes in the dataset. In forensic casework, biological samples are often challenged by various environmental factors and there is no alternative but to use those samples with compromised RNA integrity. In addition, it has been discussed that a high RNA input seems to be more important than high RIN values, since many samples with low RIN values generated high sequencing output [46].

Exploratory data analysis revealed no distinct expression patterns between the SUD and controls, nor among the three SUD subgroups. For some of the control samples, a distinct expression pattern was identified, which led to a more detailed examination of these cases and finally to a further division of the control samples into two subgroups “*fast death*” and “*other*”. While the “*fast death*” group consisted of cases who had died e.g. in a car accident or by suicide, the group “*other*” included heart-healthy controls with diseases such as meningitis or pneumonia. The latter is a very heterogeneous group, and we cannot exclude that the reported diseases had some effect on the cardiac transcriptome, which could be the reason why these cases clustered with the SUD cases and not with the “*fast death*” controls. In general, this subgroup division was only done to control for unwanted variation in the data, as our focus was on investigating what separates SUD cases from heart-healthy individuals. A very interesting case is SUDS104, which showed an expression pattern different from the rest of the SUD and control cases. The deceased had already been diagnosed with a specific cardiac disease (SQTS) during his lifetime and in addition, he was very young (below 18) at the time of death compared to most other cases. Since the differences in the expression pattern could not be attributed to the young age (no clustering according to age, see Supplementary Figure S2D), the separation might be explained by a very specific expression pattern caused by a cardiac arrhythmia.

By comparing the SUD and control cases, DESeq2 identified 1,676 significantly differentially expressed genes, the majority of which were up-regulated (1,093 genes). This may be because genes were activated as part of a compensatory or regulatory mechanism, or in response to stress [47]. Among the differentially expressed genes, 42 were on our list of candidate genes associated with cardiovascular diseases. The most interesting differentially expressed cardiac genes are *CACNA2D1*,* CASQ2*,* CDH2*,* DSC2*,* DSP*,* GJA5*,* GPD1L*,* KCNE3*,* LAMA4*,* MYOZ2*,* NEXN*,* SCN4B*,* SDHA*,* SLMAP* and *SNTA1*, as mutations in these genes have been reported in ion channelopathies or cardiomyopathies [48, 49]. In addition, reduced expression level of one of these genes, *NEXN*, in combination with a mutation in the promoter region has already been identified in a SADS case [20]. *NEXN* encodes the actin filament-binding protein nexilin, which connects the actin cytoskeleton to the plasma membrane and is predominantly expressed in cardiomyocytes and skeletal muscle cells [50]. Global knockout of NEXN in mice has been reported to cause a rapidly progressive cardiomyopathy leading to lethality shortly after birth [51]. In humans, heterozygous *NEXN* mutations have been reported to cause dilated or hypertrophic cardiomyopathy with incomplete penetrance and variable age of onset [52–54]. Furthermore, biallelic loss-of-function variants in *NEXN* have been reported in three cases of lethal fetal cardiomyopathy [54–56], indicating that nexilin plays an important role in both heart development and maintenance of cardiac architecture in adults.

Pathway analysis of the differentially expressed genes between SUD cases and controls showed that most of them were downregulated and involved in amide/peptide biosynthetic processes and fatty acid metabolism. Most of these pathways are primarily active in mitochondria, which play a key role in energy production, but are also involved in free radical production, calcium homeostasis, cell survival, cell apoptosis and necrosis [57]. Due to the high energy demands of the heart, mitochondria are abundant in cardiomyocytes, whereas ATP-production is primarily dependent on fatty acids [58]. Mitochondrial dysfunction, e.g. caused by impaired fatty acid metabolism, is considered a crucial contributory factor to cardiac pathology and has been implicated in the development of heart failure [59–61]. Therefore, a downregulation of these pathways could point to a compromised energy supply in the heart of SUD cases, leading to an acute impaired cardiac function and heart failure. The few upregulated pathways were mainly assigned to intra- and extracellular signaling pathways, such as neuroactive ligand-receptor interaction, cell surface receptor signaling, and transmembrane signaling receptor activity, which have been described in the pathogenesis of chronic heart failure [62]. Therefore, future studies could focus on analyzing postmortem metabolomic and proteomic changes in the heart to deepen our understanding of the various modifications and biochemical alterations in the here reported pathways [63, 64].

It is well known that SUD cohorts are very heterogeneous, with different underlying pathophysiological mechanisms, and therefore most probably not optimally suited for transcriptome analysis. We further divided our SUD cases into more well-defined SUD subgroups based on the autopsy reports, heart morphology and histopathological findings. However, for the three subgroups “*primary normal*”, “*primary cardiomyopathy*” and “*secondary condition*”, no distinct separation between these individual subgroups and the controls was observed. Nevertheless, it was apparent that “*primary cardiomyopathy*” exhibited the most pronounced differences. The number of cardiac related genes significantly differentially expressed between “*primary cardiomyopathy*” cases and controls was higher than between all SUD cases and controls. On the other hand, the “*secondary condition*” group showed the least differences. It appears that the “*primary cardiomyopathy*” samples are the driving force behind the transcriptomic differences observed between SUD cases and controls. We are aware that the sample sizes differ among the SUD subgroups (from *n* = 16 for “*primary normal*” to *n* = 7 for “*secondary condition*”), and we expect to observe fewer DE genes for smaller groups due to less statistical power. In addition, the age distribution is different between the “*primary normal*” (mean age = 28.9 ± 11.7 years) and the other two subgroups (“*primary cardiomyopathy*”: mean age = 35.9 ± 11.9 years, and “*secondary condition*”: mean age = 36.5 ± 8.04 years), which is another point that should be considered. Overall, we conclude that the SUD subgroups are still too heterogeneous to see distinct expression patterns between them and the controls. Consequently, more well-defined larger subgroups would be needed to identify specific gene expression patterns and pathways related to cardiac diseases. For example, a systematic exploration amongst primary cardiomyopathies (ACM, HCM, DCM and restrictive cardiomyopathy) identified several metabolic pathways that are significantly dysregulated in cardiomyopathies, with specific pathways either up- or downregulated, depending on the type of cardiomyopathy [65]. Beside the heterogeneity of the SUD cohort, it is important to remember that cardiac diseases are complex disorders caused by the interaction of different genomic factors, modifier genes and environmental factors [66, 67], making it difficult to identify specific expression patterns.

When focusing on the top DE genes, several genes were common to the three SUD subgroups. One of these is the gene *RNF213*, which encodes the ring finger protein 213, and variants in this gene have been linked to Moyamoya disease, arterial stenosis/occlusion, atherosclerosis, ischemic stroke and intracranial aneurysms [68]. Another gene is *EGFL7* (epidermal growth factor-like protein 7), a highly conserved secreted extracellular matrix binding factor that is uniquely expressed by endothelial cells [69]. EGFL7 expression is upregulated during physiological angiogenesis and plays a role in the repair of the microvasculature in response to vascular injury or hypoxic environment [70]. Another interesting gene is *HSPG2*, which encodes the heparin sulfate proteoglycan 2 protein, that is a key component of the vascular extracellular matrix and is widely expressed in cardiac fibroblasts. Upregulation of *HSPG2* has been reported in DCM patients, and increased expression in these patients has been strongly associated with immune activation, cardiac fibrosis and heart failure [71]. Furthermore, NADH dehydrogenase (ubiquinone) 1 alpha sub-complex 4 like 2 (*NDUFA4L2*) is involved in reducing mitochondrial activity and oxygen consumption, and hypoxia-induced *NDUFA4L2* expression has been observed in mouse neonatal cardiomyocytes [72].

Our SUD cohort included four children (aged 6 (twice), 8, and 11 years) and one 17-year-old adolescent, but no distinct separation was observed between these cases and the rest of the SUD cohort. It has been reported that RNA expression shows quite different patterns in the heart of young children compared to adult hearts [73]. Animal models have shown that fetal and neonatal gene expression differs from that of adult hearts, and molecular findings in explanted hearts from children have demonstrated age-related differences [74]. In addition, a recent study of HCM patients showed that some pathways such as actin filament organization or protein synthesis pathways are shared between pediatric and adult hypertrophic cardiomyopathy, whereas mitochondrial function is specifically dysregulated in adult HCM and the inhibition of cardiac developing networks is characteristic of pediatric HCM [75]. For our cohort, we concluded that the sample size of four children is far too small to distinguish the transcriptome pattern between children and adults.

Postmortem exome sequencing in our SUD cohort had identified 13 pathogenic/likely pathogenic variants in the coding part of genes associated with cardiac diseases in seven of the here analyzed cases [8, 21]. However, the transcriptome data did not reveal any differences in the expression levels of samples with a variant compared to those without, suggesting that the identified variants likely affect the function of the gene product without altering its expression level. A more promising approach would be to focus on changes in the transcription levels caused by variants that are not or only partially covered by exome sequencing, such as variants located in the promoter region or UTR splice-site variants, which was beyond the scope of this study. For example, RNA sequencing of a sudden death patient revealed an abnormal *LDB3* splicing pattern, which is linked to myotonic dystrophy type 1 (DM1), whereas previously performed clinical exome sequencing had identified a variant in *RBM20* of unclear significance [76]. DM1 is caused by expanded unstable CTG repeats in the 3’ untranslated region of *DMPK*, leading to dysregulation of RNA-binding proteins and preventing correct splicing of several other mRNAs. Sequencing of the non-coding part of *DMPK* in the patient’s sample confirmed an increased number of CTG repeats and therefore the diagnosis of DM1, suggesting that RNA sequencing could be helpful in determining the molecular diagnosis of sudden death in cases where the exome sequencing data are inconclusive.

There are some points to consider for this study. First, the transcriptome analysis was only performed for one region of the heart (left ventricle), but depending on the underlying cardiac diseases in the SUD cases, other regions of the heart might be affected. In addition, we are aware that some of our cases were assumed to be SUDEP and therefore the transcription pattern of the brain should be investigated as well. This was not possible as no brain tissue was collected during autopsy. Second, while RNA-Seq studies of heart tissue can provide important insights into regional and pathological differences in tissue-level expression, cell subtype-specific expression profiles cannot be captured with this technology. As the heart is composed of four major cell types, namely cardiomyocytes, cardiac fibroblasts, smooth muscle cells and endothelial cells, single-cell RNA sequencing (scRNA-seq) represents a powerful tool to identify transcriptional signatures at the individual cell level and to investigate disease-associated changes in the cell states [77, 78]. Third, RNA-Seq analysis should also focus on other types of RNA, such as microRNA, long non-coding RNAs or circular RNAs, which could serve as biomarkers to discriminate the cause of cardiac death [79, 80]. Fourth, larger cohorts of well-defined SUD subgroups would be required to identify specific expression patterns for specific heart diseases.

To our knowledge, this is the first transcriptome study in a larger cohort of sudden unexplained death cases. Although the investigated SUD subgroups are too heterogeneous to identify specific pathways associated with a particular cardiac disease, we conclude that transcriptome analysis could help to provide additional insights into the pathological mechanisms of a sudden death event.

## Electronic supplementary material

Below is the link to the electronic supplementary material.

Supplementary Material 1

Supplementary Material 2
