# Supplementary material for: Exploring transcriptomic signatures in sudden unexplained death (SUD) cases
Source: Int J Legal Med. 2025 Feb 21;139(4):1477–93. doi: 10.1007/s00414-025-03414-4 (PMC12170759; doi:10.1007/s00414-025-03414-4)
Supplement: Supplementary file 2 — Supplementary Material 2 [file 414_2025_3414_MOESM2_ESM.pdf]

Supplementary figures

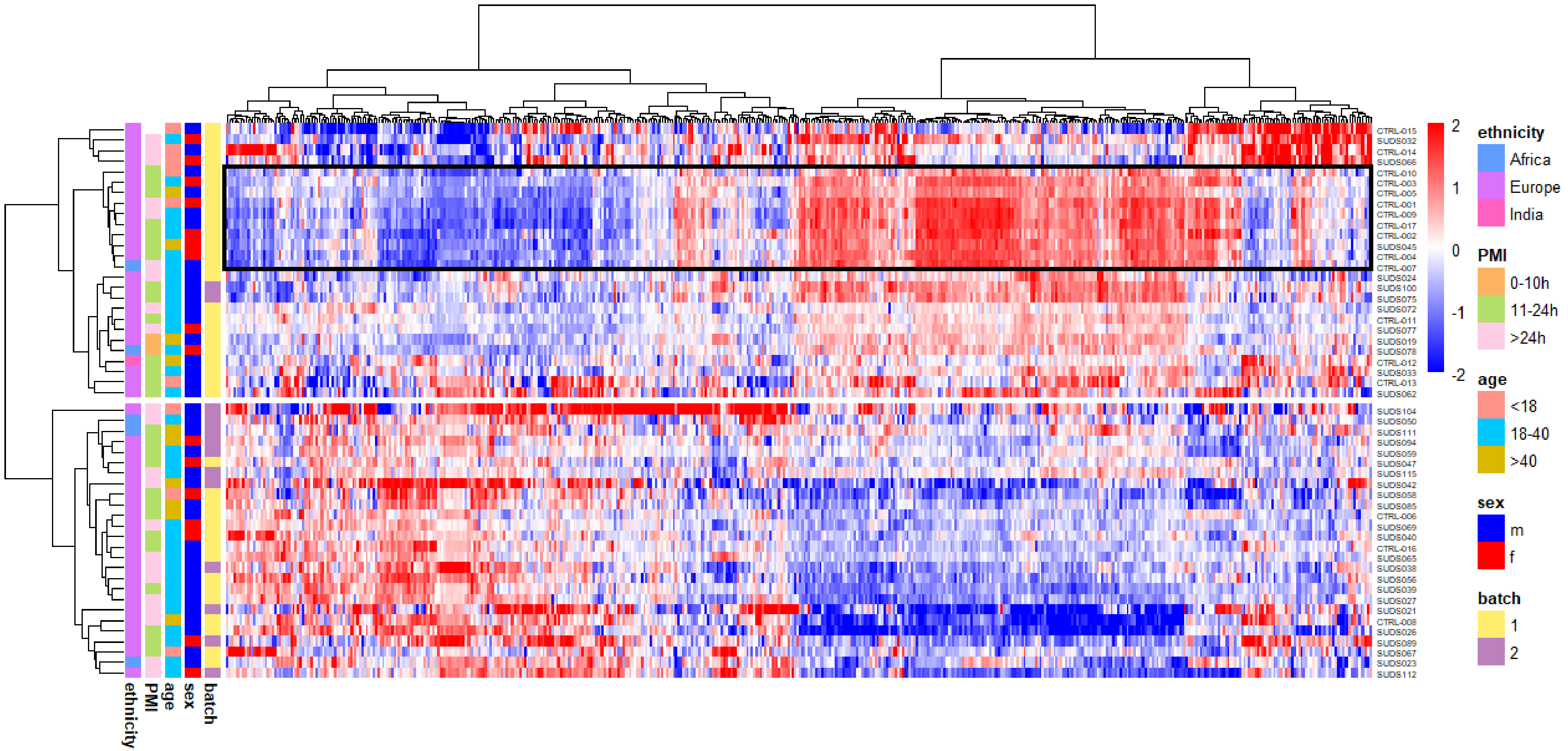

Figure S1 Heatmap of the top 500 genes with the highest variance. The black frame displays a cluster of samples with a distinct expression pattern; including nine "*fast death*" control samples and one SUD case. No clustering according to ethnicity, postmortem interval (PMI), age, sex or batch can be observed. Red-blue scale: red represents upregulation and blue downregulation. *f* female, *m* male, *PMI* postmortem interval

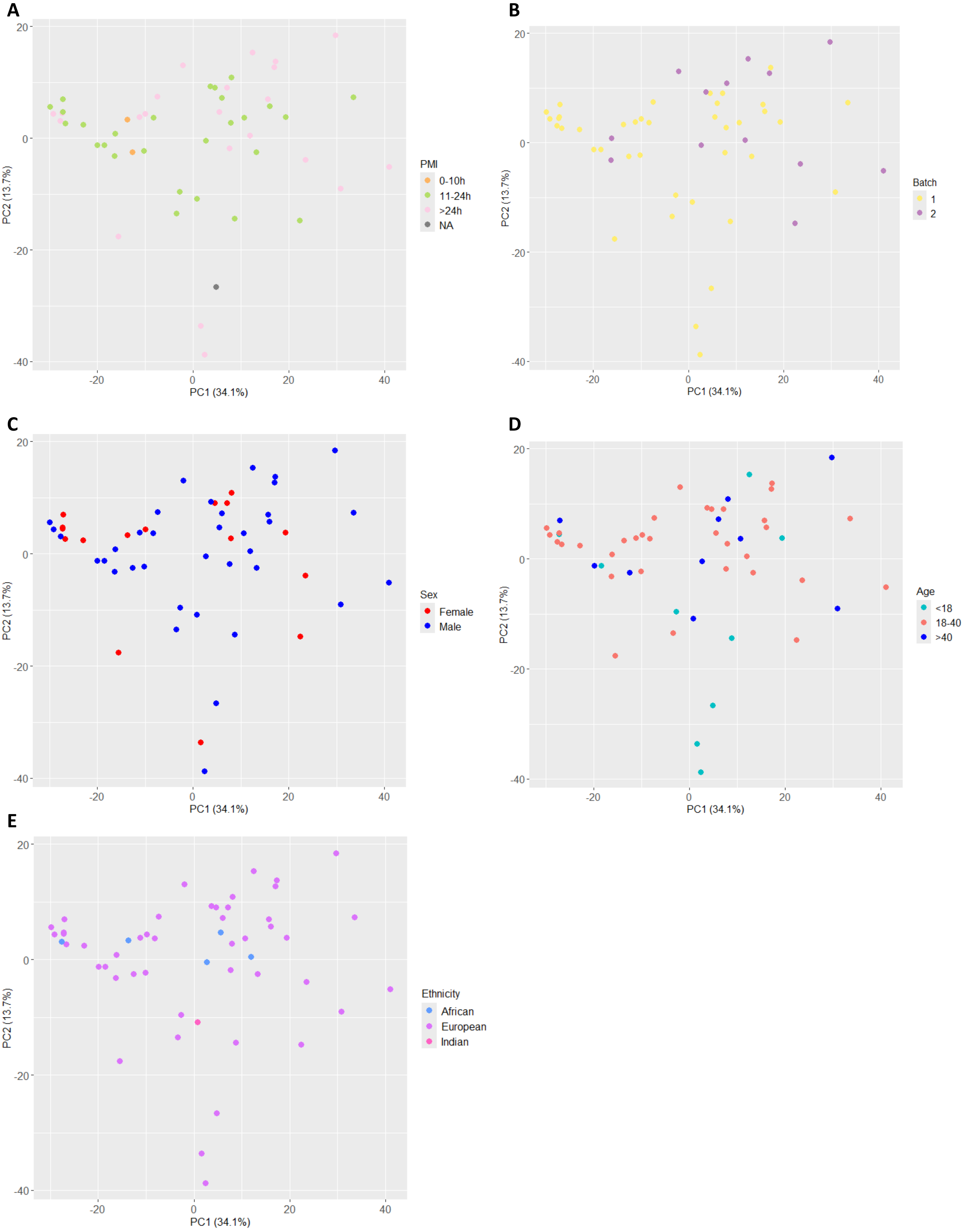

Figure S2 PCA plot of the top 500 gene expression profiles of 35 SUD and 17 control cases. The samples are colored according to (A) postmortem interval (PMI), (B) batch, (C) sex, (D) age and (E) ethnicity.

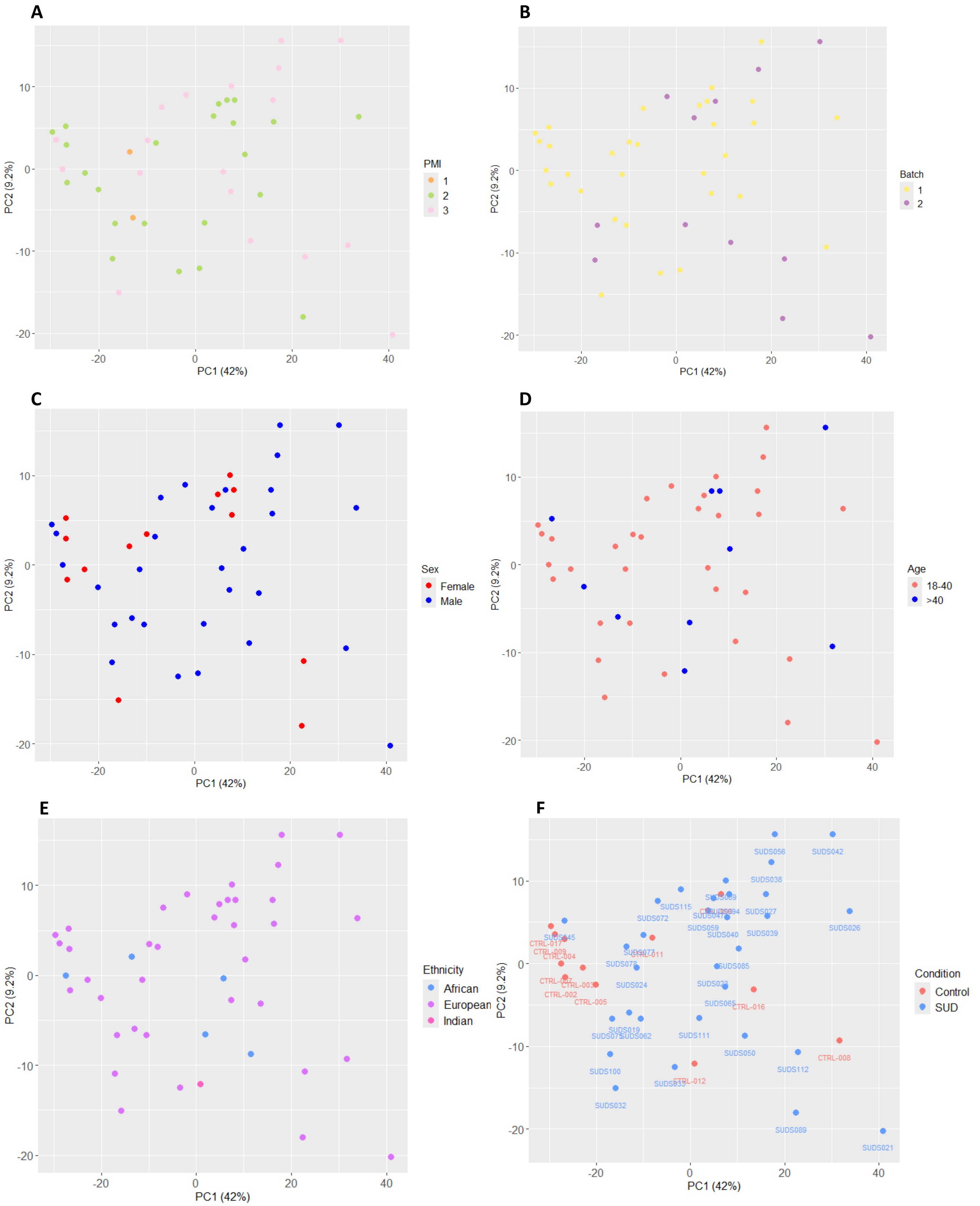
A
PC2 (9.2%)
PC1 (42%)
PMI
1
2
3
B
Batch
1
2
C
Sex
Female
Male
D
Age
18-40
>40
E
Ethnicity
African
European
Indian
F
Condition
Control
SUD
SUDS056
SUDS042
SUDS038
SUDS115
SUDS072
SUDS059
SUDS027
SUDS040
SUDS039
SUDS026
SUDS085
SUDS078
SUDS024
SUDS023
SUDS065
CTRL-016
CTRL-017
CTRL-004
CTRL-011
CTRL-002
CTRL-005
SUDS019
SUDS062
SUDS111
SUDS050
SUDS112
CTRL-008
SUDS100
CTRL-012
SUDS032
SUDS089
SUDS021

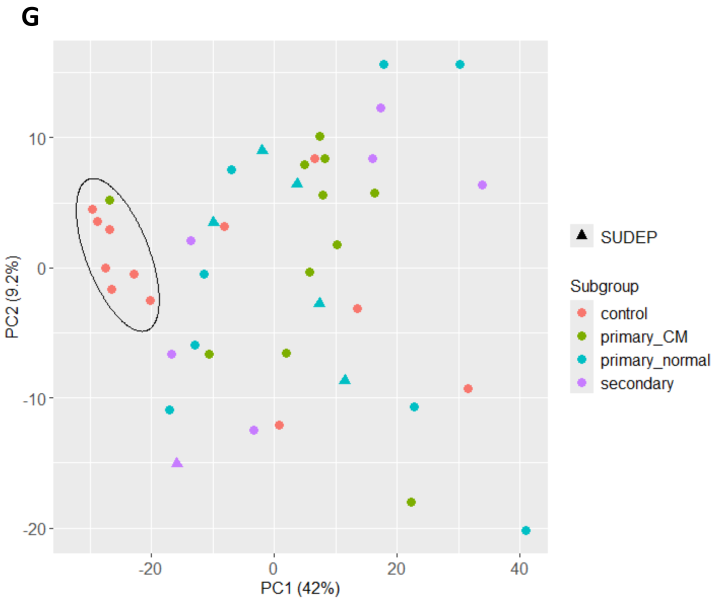

Figure S3 PCA plot of the top 500 gene expression profiles of 31 SUD and 12 control cases above the age of 18. The samples are colored according to (A) postmortem interval (PMI), (B) batch, (C) sex, (D) age, (E) ethnicity, (F) conditions and (G) subgroups.

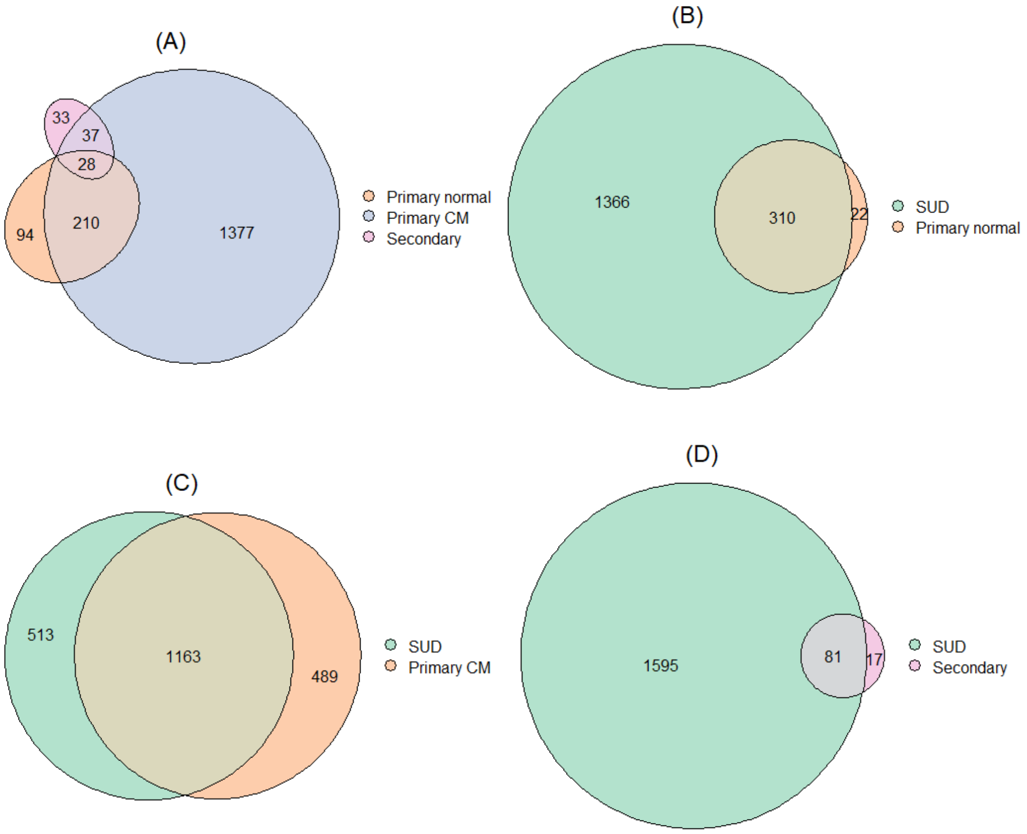

Figure S4 Overlapping DE genes between (A) SUD subgroups, (B) SUD and “*primary normal*”, (C) SUD and “*primary cardiomyopathy*” and (D) SUD and “*secondary condition*”.

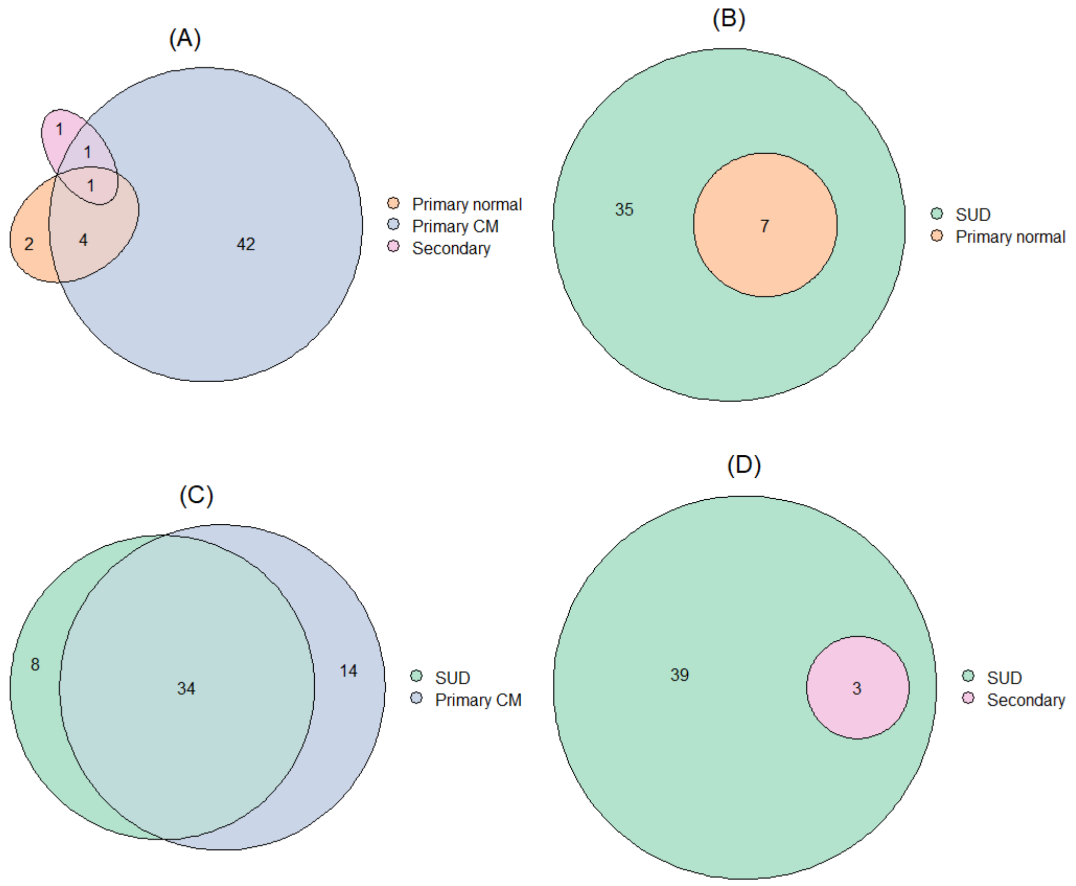

Figure S5 Overlapping cardiac genes between (A) SUD subgroups, (B) SUD and "primary normal", (C) SUD and "primary cardiomyopathy" and (D) SUD and "secondary condition".

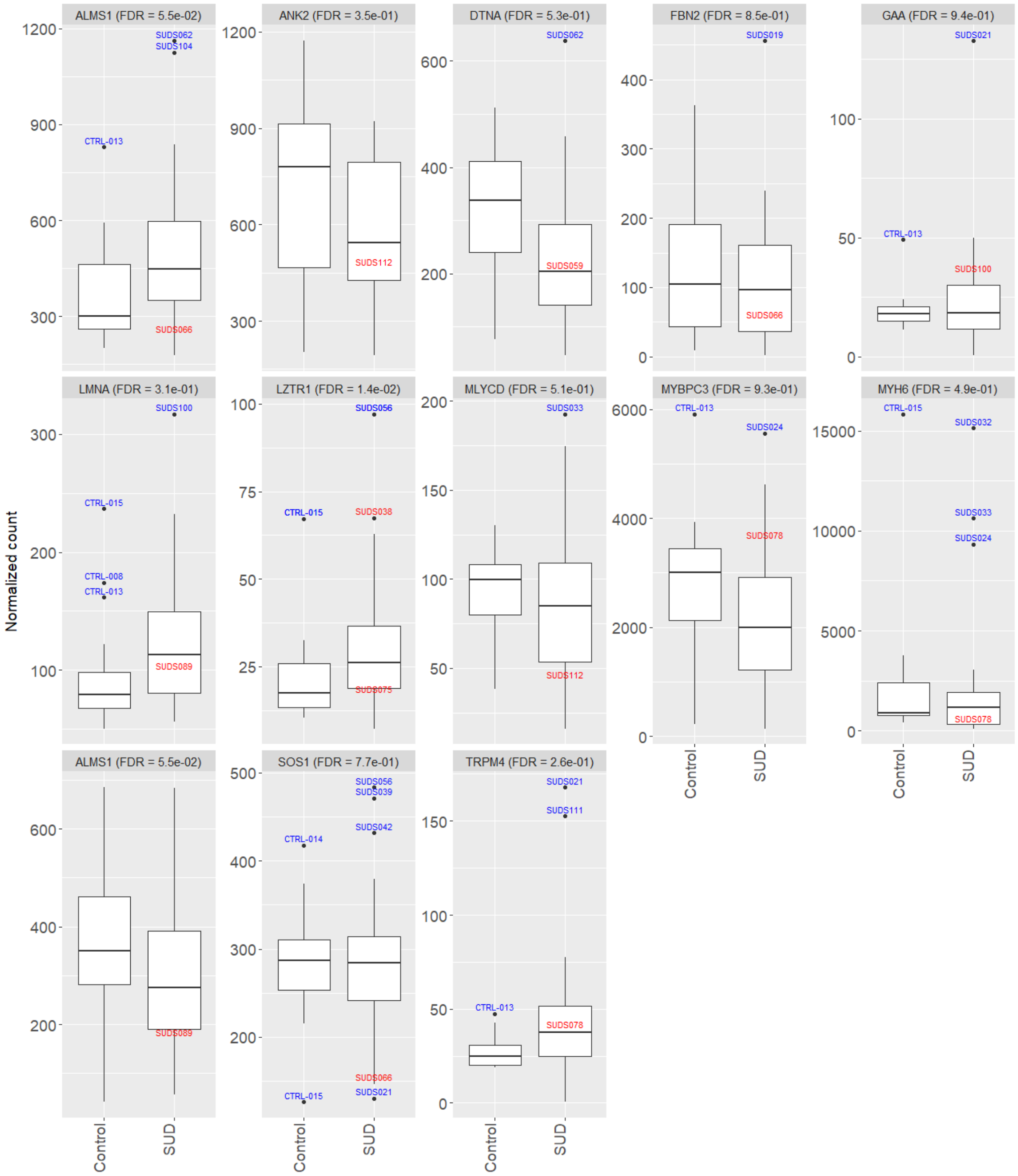

Figure S6 A boxplot displaying normalized read counts for 13 genes with pathogenic or likely pathogenic variants. Samples labelled in red indicate where variants were found, while blue samples denote outliers within the SUD and control samples, which vary across the genes.
